# Hematopoietic Stem Cell Transplantation in Adult Sickle Cell Disease: Problems and Solutions

**DOI:** 10.4274/tjh.2014.0311

**Published:** 2015-08-01

**Authors:** Hakan Özdoğu, Can Boğa

**Affiliations:** 1 Başkent University Faculty of Medicine, Department of Internal Medicine, Division of Hematology, Adana, Turkey

**Keywords:** Sickle cell disease, Hematopoietic stem cell transplantation, graft-versus-host disease, Graft rejection, Conditioning

## Abstract

Sickle cell disease-related organ injuries cannot be prevented despite hydroxyurea use, infection prophylaxis, and supportive therapies. As a consequence, disease-related mortality reaches 14% in adolescents and young adults. Hematopoietic stem cell transplantation is a unique curative therapeutic approach for sickle cell disease. Myeloablative allogeneic hematopoietic stem cell transplantation is curative for children with sickle cell disease. Current data indicate that long-term disease-free survival is about 90% and overall survival about 95% after transplantation. However, it is toxic in adults due to organ injuries. In addition, this curative treatment approach has several limitations, such as difficulties to find donors, transplant-related mortality, graft loss, graft-versus-host disease (GVHD), and infertility. Engraftment effectivity and toxicity for transplantations performed with nonmyeloablative reduced-intensity regimens in adults are being investigated in phase 1/2 trials at many centers. Preliminary data indicate that GVHD could be prevented with transplantations performed using reduced-intensity regimens. It is necessary to develop novel regimens to prevent graft loss and reduce the risk of GVHD.

## Abstract (tr)

Günümüzde hidroksiüre kullanımı, enfeksiyon proflaksisi ve destek tedavileriyle ilgili gelişmelere rağmen orak hücre hastalığına bağlı organ hasarları engellenememekte, adölesan ve genç yetişkinlerde hastalık ilişkili mortalite %14’lere kadar ulaşmaktadır. Hematopoietik kök hücre transplantasyonu, orak hücre hastalığı tedavisinde tek küratif tedavi yaklaşımıdır. Myeloablatif allojenik hematopietik kök hücre transplantasyonu orak hücre hastalığı olan çocuklar için küratiftir. Güncel veriler çocuklarda transplant sonrası uzun dönem hastalıksız yaşam oranının %90 ve toplam yaşam oranının %95 civarında olduğunu göstermektedir. Erişkinde hazırlama rejimleri organ hasarları nedeniyle fazla toksiktir. Ayrıca bu küratif tedavi yaklaşımının donör bulma güçlüğü, transplant ilişkili mortalite, graft kaybı, graft-versus-host hastalığı (GVHH), infertilite gibi nedenlerle sınırlamaları vardır. Güncel olarak erişkinde toksisitesi azaltılmış hazırlama rejimleri (myeloablatif olmayan indirgenmiş yoğunluklu) ile yapılan transplantların faz 1/2 çalışmalar ile graft yerleşmesi üzerine etkinliği ve toksisitesi birçok merkez tarafından araştırılmaktadır. Ön veriler, toksisitesi azaltılmış rejimler ile yapılan nakillerde başarılı olarak GVHH’nin engellenebildiğini göstermiştir. Bununla birlikte yüksek graft kaybı riski devam etmektedir. Bu yüzden orak hücre hastalarında uygulanabilen, GVHH riski azaltılmış ve graft kaybının engellenebildiği yeni rejimlerin geliştirilmesi gereklidir.

## INTRODUCTION

### Disease Overview

Sickle cell disease (SCD) is a homozygous hemoglobin S disease characterized by chronic anemia and tissue injury. Annually, approximately 300,000 babies are born with SCD worldwide. One in every 600 African-Americans in the United States is affected by the disease [1]. Central to the pathogenesis of SCD is a mutation leading to abnormal polymerization of hemoglobin and formation of the characteristic sickle shape as a response to deoxygenation [2,3]. Microvascular ischemia and endothelial activation are responsible for tissue injury.

To date, drug therapy for SCD is limited to fetal hemoglobin-stimulating medicines and nonnarcotic and narcotic agents. Use of antisickling agents is not standard, while experience with gene therapy is insufficient. Allogeneic stem cell transplantation is a curative intervention, but it carries a high risk of morbidity and mortality. The development of transplant techniques seems to give better results. In this review, we aim to highlight questions related to transplantation procedures and to discuss our proposals.

### Current Reasons for Transplantation

Current reasons for accepting the risk of transplantation are summarized below. First, the clinical manifestations and complications are dramatic in SCD patients. Second, despite their high costs, no treatment protocol is for protecting from complications. Third, transplantation techniques and patient care have evolved over time.

### Manifestations and Complications of Sickle Cell Disease

SCD is characterized by hemolytic anemia, painful vaso-occlusive crisis, stroke, avascular necrosis, pulmonary hypertension, susceptibility to infections, renal failure, and thrombosis. Consequently, life expectancy is decreased. Neurologic complications develop in 27% of children with SCD and acute chest syndrome in 25% [4,5]. These tissue injuries significantly influence quality of life in most patients.

### Frontline Treatment Options and Disease Course

The current, treatment modalities for SCD include fluid replacement, pain control using opioids and analgesics during vaso-occlusive crises, oral hydroxyurea, transfusion, and chelating therapy. Hydroxyurea is effective for reducing the frequency of painful crises and recommended for adults with recurrent episodes of acute chest syndrome [6,7]. Maintaining sickle hemoglobin levels of less than 30% by transfusion may prevent or control adverse events associated with SCD [8,9]. However, these complications may develop despite hydroxyurea use and regular blood transfusions [4,5]. Painful ischemic episodes lead to irreversible sequelae by affecting bones, joints, muscles, and the heart, intestines, kidneys, and eyes. Mean survival is 40 years in developed countries despite advanced supportive care [10]. At our institute, the mean age of patients who died within the last 10 years was 30 years (unpublished data).

### Cost

The disease also has high treatment costs. A study using data from the Florida Medicaid program showed a healthcare cost per patient per month of $1389, with significant increases in SCD-related costs from $892 in the 0-9-years cohort to $2562 in the 50-64-years cohort [11]. The cost of chronic transfusions and chelating therapy alone is $40,000 per patient per year in the United States [12,13].

### Allogeneic Stem Cell Transplantation

Hematopoietic stem cell transplantation (HSCT) is a unique curative therapeutic approach in SCD [14,15,16]. HSCT may improve central nervous system (CNS), pulmonary system, and pain symptoms by stabilizing organ functions [17,18]. Overall survival was reported to be 90%, disease-free survival 82%-100%, graft rejection 8%-18%, and transplant-related mortality (TRM) 4%-14% in transplantations performed mainly with myeloablative conditioning regimens with fully HLA-matched donors in various countries [15,19,20,21,22]. However, this curative treatment approach has some limitations, such as difficulties to find donors, TRM, graft loss, graft-versus-host disease (GVHD), and infertility.

### Indications for Transplantation

#### Problem:

Although HSCT has a curative potential in this nonmalignant disease, it is difficult to determine in which patients the risk of the procedure is acceptable.

### Current HSCT indications are summarized in Table 1.

Extreme variation in the clinical phenotypes of SCD patients makes it difficult to predict the course of the transplantation procedure. However, certain clinical and laboratory factors may predict transplant-associated problems. These factors are renal insufficiency, age >16 years, hepatic function abnormalities, and high inflammation [5,17,22,23,24]. In addition, alloantibodies against erythrocytes and HLA antigens and excess iron load develop due to transfusions in the vast majority of patients [25]. Transcranial Doppler ultrasound was shown to be effective for determining the severity of the disease and stroke risk in children. Starting blood transfusion therapy early reduces the risk [26]. Unfortunately, no reliable method is available to predict which patients would develop stroke.

On the other hand, hydroxyurea reduces the frequency and severity of vaso-occlusive crises and acute chest syndrome crises. However, organ injuries continue to occur despite hydroxyurea and current supportive therapies, and mean survival of patients is about 40 years [4,27]. The presence of organ injuries is associated with increased mortality and morbidity.

#### Proposed solution:

Adult SCD patients have generally been excluded from myeloablative bone marrow transplantation trials because of anticipated excess morbidity and mortality resulting from accumulated disease-related end-organ damage. Rational thought requires applying a curative treatment (i.e. HSCT) in the early period before organ injuries that significantly reduce the life span develop.

### Donor

#### Problem:

The likelihood of finding an HLA-identical sibling donor is low for SCD patients.

In the United States, the estimated number of homozygous SCD patients is around 70,000-100,000 and the overall number of patients who have indications for transplantation is around 5000-7000. A total of 1200 transplantations for SCD were reported in 1986-2013 according to Center for International Blood and Marrow Transplant Research (CIBMTR) and European Society for Blood and Marrow Transplantation (EBMT) records. These rates may be stated to be much lower than needed and expected (Table 2). In a study by Hsieh et al., 24 out of 112 adult patients (21%) had suitable donors for transplantation. However, the procedure could only be performed in 10 patients (8.9%) [28].

The vast majority of healthy HLA-identical sibling donors are hemoglobin S carriers. Although there is evidence that peripheral stem cell mobilization with G-CSF is safe in hemoglobin S carrier donors, it should be remembered that G-CSF administration could lead to severe and sometimes fatal sickle cell crisis in carrier donors [29,30,31]. Multiorgan failure was reported in 2 from 11 cases and in 1 case it was fatal. Four people who developed complications required hospitalization. Multiorgan failure and sickle cell crisis were reported to be independent of leukocyte count and G-CSF dose (2.5-10 μg/kg/day), but they seem to be associated with hemoglobin S concentration [29,30,31]. Erythrocyte exchange or erythrocyte transfusion may be required to reduce the level to <30% prior to mobilization. However, there are no data for plerixafor mobilization in hemoglobin S carrier donors. Theoretically, plerixafor might be safer than G-CSF for mobilization as it does not trigger hyperleukocytosis and bone marrow hyperplasia [32].

Severe pain may begin 1 h after apheresis without mobilization due to transfusion of the erythrocytes, which wait hypoxically in the pulley of the apheresis device. Bone marrow harvesting is safe under local or general anesthesia with careful perioperative management. However, the difficulty in collecting an adequate amount of stem cells for an adult should be remembered. On the other hand, no technical difficulties for cryopreservation were reported in sickle cell carriers who could easily tolerate the procedure [28,29,30,31,33].

#### Proposed solution:

Due to the abovementioned problems, donors who are not carriers should preferably be selected in the interest of donor safety. The hemoglobin S level should be decreased to <30% if the donor is a carrier. Bone marrow should be harvested, if possible. Plerixafor should be preferred for mobilization of carrier donors and the rinse-back procedure should be avoided in apheresis procedures.

Overall, less than 25% of SCD patients have a healthy HLA-identical relative donor. HLA-identical unrelated donors can be found for only a small proportion of patients [34]. A trial of unrelated cord blood transplantation (CBT) was terminated early due to high risks of rejection and GVHD [35]. On the other hand, almost all patients have a haploidentical relative donor. Bolanos-Meade et al. showed that no patients who underwent mismatched transplantation using cyclophosphamide developed GVHD or severe immunodeficiency complications [36]. However, the risk of graft rejection is high in these patients as they are immunocompetent and have proliferative bone marrow. Conditioning regimens are designed to give a low GVHD risk. This problem may be overcome by further developing conditioning regimens (see conditioning regimens below).

### Stem Cell Source

#### Problem:

The ideal stem cell source is not known.

#### Bone marrow:

Bone marrow should be the preferred as the stem cell source due to the low risk of chronic GVHD (cGVHD). However, HLA-matched donors can be found for only a small proportion of patients. G-CSF-mobilized marrow has been used in the haploidentical (family donor) setting. The use of G-CSF-primed bone marrow grafts to reduce graft loss has been reported [36]. The difficulty in collecting a sufficient amount of stem cells in adults should be considered. In addition, there is evidence that donors of African origin have lower blood counts and reduced numbers of marrow progenitor cells [37].

Haploidentical bone marrow transplantation with posttransplant cyclophosphamide has been applied in patients with hematological malignancies for several years. The Johns Hopkins Group reported that this approach is a feasible and effective treatment with acceptable toxicity [38]. Evidence on the potential use of bone marrow from haploidentical related donors has increased in patients with SCD. Bolanos-Meade et al. reported promising results with haploidentical nonmyeloablative bone marrow transplantation. They reported durable engraftment and acceptable toxicity with no morbidity and mortality in their series [36].

#### Peripheral stem cells:

They can easily be collected without general anesthesia in the outpatient setting. Their application is less traumatic and has advantages such as less need for transfusion, faster engraftment, less need for platelet suspension, and shorter hospital stays. Although peripheral stem cell products with a 10-fold higher T-cell count increase the risk of cGVHD, their engraftment-facilitating effect should be considered.

#### Cord blood:

This stem cell source has some limitations, such as difficulty to find an HLA-identical donor, GVHD, and graft rejection, despite data showing successful treatment of patients by CBT. A study from the Eurocord Cooperative Group, analyzing the outcome in 44 patients who had SCD or thalassemia major and were treated by CBT with a sibling donor, reported no fatal transplantation-related complications, suggesting that CBT with a related donor is a safe treatment for hemoglobin disorders [38]. However, CBT with matched unrelated donors will likely not be applicable to sufficient numbers of SCD patients because of a lack of suitable donors in national and worldwide registries. CBT with mismatched unrelated donors, although more feasible for pediatric patients based on the availability of 4/6 HLA-matched cord blood units, appears to be associated with a greater risk of graft rejection and GVHD on the basis of the limited data available [39]. Drawbacks such as increased rates of graft rejection, the fixed cell dose, delayed immune reconstitution, and TRM have deterred unrelated cord transplantation efforts [39]. Furthermore, CBT for adults will be limited by the necessary total nucleated cell count per kilogram of body weight for engraftment to occur and the difficulty in achieving this goal in adult patients. Double umbilical cord blood allogeneic transplantation with reduced-intensity conditioning is increasingly used in adults lacking a suitable related or unrelated donor [40]. However, the studies of expanded and double cord blood are inadequate.

#### Proposed solution:

Current data indicate that peripheral stem cells mobilized with G-CSF could be a suitable stem cell source for patients with SCD [32]. If the donor is a carrier, decreasing hemoglobin S level below 30% of total hemoglobin concentration might be recommended for a safer mobilization process and apheresis procedure. Hematopoietic and lymphoid reconstruction is rapidly achieved through replacement of T cells by donor peripheral stem cells. Bone marrow from haploidentical donors might be an alternative to matched related or unrelated donors, but this needs more research [40]. Mesenchymal stem cell co-infusion may be considered to prevent GVHD and/or cGVHD and graft loss [41,42,43].

### Conditioning Regimens

#### Problem:

Engraftment efficacy and toxicity for transplantation performed with nonmyeloablative reduced-intensity conditioning regimens are currently being investigated in phase I/II trials at many centers. Preliminary data indicate that GVHD could be prevented by nonmyeloablative reduced-intensity regimens. However, the risk of graft loss remains high.

Allogeneic bone marrow transplantation performed with an HLA-matched donor using a myeloablative-conditioning regimen can cure symptomatic children with SCD. The cure rate was reported to be 85%, the rate of TRM 7%, and the rate of graft rejection 8% in studies including approximately 200 patients [16,38,44]. The results were improved by adding ATG to the conditioning regimen, which gave a cure rate of 95% (Table 3) [45]. Adult SCD patients have to be excluded from most studies using ablative regimens due to end-organ injury-related morbidity and mortality in most patients, despite positive results in childhood.

In the past, nonmyeloablative bone marrow transplantation strategies developed to cure SCD patients with organ dysfunction gave disappointing results [46,47]. Transplantations done with nonmyeloablative regimens were not as successful as myeloablative transplantations. The main reasons for this included the recipient’s not being fully immunocompetent, having proliferative bone marrow, and lack of a GVHD effect. Recently, inconsistently with previous studies, several small patient series have shown promising results [28,36,48,49]. Therefore, HSCT following a reduced-intensity conditioning nonmyeloablative regimen emerged as a potential treatment option (Table 4).

In general, small proportions of engrafted donor red cells seem to be sufficient for clinical control of the disease in SCD patients [50]. Hsieh et al. reported that they achieved permanent engraftment in 26/30 patients and that no patients developed acute GVHD (aGVHD) or cGVHD with a nonmyeloablative-conditioning regimen using alemtuzumab (Campath) and low-dose total-body irradiation (TBI). The mean donor T-cell level was 48% and the myeloid chimerism level was 86%. Fifteen engrafted patients discontinued immunosuppression medication with continued stable donor chimerism and no GVHD [49]. In this study, sirolimus (rapamycin) was selected as the immunosuppressive agent instead of cyclosporine. Differently from calcineurin inhibitors, sirolimus does not block T-cell activation, but it inhibits T-cell proliferation. The activated but nonproliferated T cells become anergic, resulting in T-cell tolerance. Sirolimus accelerates the differentiation of regulatory and helper T cells and this plays a key role in immune tolerance development. Additionally, the renal toxicity of sirolimus is less than that of cyclosporine. Luznik et al. showed that high-dose cyclophosphamide given in the early posttransplantation period could lead to immune tolerance by killing proliferative alloreactive T cells and preserving nonreactive T cells. High-dose cyclophosphamide is highly toxic to lymphocytes, but hematopoietic stem cells are not affected as they are rich in the aldehyde dehydrogenase enzyme that metabolizes the drug. Posttransplantation high-dose cyclophosphamide deletes alloreactive T cells without affecting nonreactive T cells or hematopoietic stem cells, thereby reducing the risk of GVHD and enabling immune reconstitution [38]. Bolanos-Meade et al. achieved bone marrow transplantation in adult SCD patients with 14 haploidentical relative donors and 3 matched sibling donors using a nonmyeloablative-conditioning regimen containing cyclophosphamide, ATG, fludarabine, TBI (200 cGy), and posttransplantation high-dose cyclophosphamide [36].

#### Proposed solution:

Novel regimens to reduce GVHD risk and toxicity and prevent graft loss in SCD patients need to be developed. Such regimens may include the following:

#### ATG-Fresenius:

The drug used in preliminary studies is referred to as ATG-thymoglobulin (rabbit) in the literature and its half-life is about 12 h. ATG-Fresenius (rabbit) has a longer half-life (4-10 days) and would provide a significant advantage for preventing graft loss and GVHD.

#### Busulfex (busulfan):

Added to conditioning regimens at microablative doses considering organ injuries. It would open spaces in the bone marrow and help to prevent graft loss.

#### Treosulfan:

Used safely and effectively instead of busulfan in nonmyeloablative regimens in thalassemia patients due to its lower toxicity [51]. However, there is no experience of its use in SCD.

#### Fludarabine:

Used in nonmyeloablative-conditioning regimens in pediatric and adult SCD patients with tissue injuries [52].

#### Sirolimus:

An immunosuppressive agent with an immunotolerogenic effect [53]. It is superior to cyclosporine for providing engraftment without GVHD and reducing posterior reversible encephalopathy syndrome incidence [28,49].

#### Posttransplantation cyclophosphamide:

Posttransplantation high-dose cyclophosphamide, which is successfully used in haploidentical transplantations, should be included in the conditioning regimen. This approach was first described by Luznik et al. Interestingly, while cyclophosphamide given in the early posttransplantation period kills proliferative alloreactive T cells, it preserves resting nonreactive T cells. Thus, immune tolerance occurs, GVHD is prevented, T-cell reconstitution is achieved in a short time, and the infection risk decreases [36]. As positive results were obtained in transplantations with HLA-unmatched donors in previous studies, it would also enable haploidentical transplantations. The donor pool would be larger and patients without fully matched donors would have a chance of being cured.

#### Pretransplantation immune suppression:

Administration of 2 cycles of dexamethasone (25 mg/m2/day for 5 days) to suppress T-cell functions, facilitate engraftment, decrease the GVHD risk, and facilitate the control of disease-related inflammation [54].

#### Peri- and posttransplantation low-dose steroid administration:

Is included in haploidentical protocols and would decrease potential immunologic events (GVHD, graft loss, etc.) by suppressing inflammatory cytokines (TNF-α, IL-2, and IL-6) in SCD, which is an inflammatory disease [55].

An adult patient who underwent peripheral stem cell transplantation with a conditioning regimen designed in accordance with the principles recommended above showed no transplant-related adverse events during 20 months following transplantation [56]. Another SCD patient who underwent peripheral stem cell transplantation reached day 10 months following transplantation and is being followed with no complications (unpublished data).

#### Late Complications

##### Problem: Development of late complications.

Patients with SCD are susceptible to common transplant-related late complications such as infertility, primary gonadal failure, primary hypothyroidism, insulin-dependent diabetes mellitus, osteoporosis, and cGVHD. Fertility is affected by multifactorial causes related to the disease and the procedure [57]. On the other hand, the course of SCD-related complications like stroke, pulmonary hypertension, acute chest, nephropathy, and acute vascular necrosis is an unresolved issue for adult patients. The general prediction is that these complications can improve after transplantation. Due to the absence of available data in adult patients, we accessed pediatric data showing long-term hematological improvements after HSCT with sustained engraftment. These data have supported this expectation in most studies [58,59,60]. However, if myeloablative regimens are used, data show that SCD-related organ injuries deteriorate after transplantation over time. This was attributed to the toxic effect of drugs [58,59,60].

##### Proposed solution:

Few published studies have evaluated infertility in patients who received a nonmyeloablative regimen. Therefore, semen cryopreservation or ovum/zygote cryopreservation should be recommended before transplantation. Current studies indicate that other transplant-related late complications, particularly aGVHD and cGVHD, are within acceptable limits in adults and can be managed by appropriate treatment with L-thyroxin, calcitriol, estrogen, zoledronic acid, etc. [50,58,59]. The use of nonmyeloablative reduced-intensity conditioning is mandatory for adult patients. In addition, it may require individualization of the conditioning regimens as discussed above.

Further studies are required in adults for determining whether reduced-intensity conditioning regimens may offer better outcomes after HSCT.

## CONCLUSIONS

Although results of HLA-matched sibling HSCT are encouraging, particularly in children, there are many barriers to this curative treatment option. What risk of mortality and GVHD will families accept in the early period of this benign disease? In one study, only 37% of relatives of patients accepted a 15% mortality risk; only 13% accepted a 15% mortality risk and a 15% GVHD risk [60].

Only a small proportion of patients may be candidates for transplantation due to the difficulty to find a matched sibling donor, absence of financial or psychosocial support, the family’s not accepting HSCT or the physician’s not choosing HCST as a treatment option. In one study, only 44 of 315 patients were found to have an HLA-matched sibling donor [61,62]. In addition, the likelihood of finding an unrelated fully matched donor is very low [34]. An unrelated CBT trial was terminated early due to high risks of rejection and GVHD [35]. Studies to enlarge the donor pool are required. The study of posttransplantation high-dose cyclophosphamide by Bolanos-Meade et al. is encouraging in this regard. Severe GVHD and immunodeficiency, which are seen in mismatched transplantation, were not seen in any of their patients [36].

Development of organ injuries in adult patients requires use of low-toxicity nonmyeloablative regimens for transplantation. This approach results in high rates of graft loss with current conditioning regimens. Although return of the disease is acceptable in such cases, it is one of the barriers that affect the success of transplantation.

Gene transfer to autologous hematopoietic stem cells via viral vectors and gene therapies performed via induced pluripotent stem cells might be possible solutions to the difficulties in finding a matched donor and are exciting treatment options [63,64].

## Figures and Tables

**Table 1:**
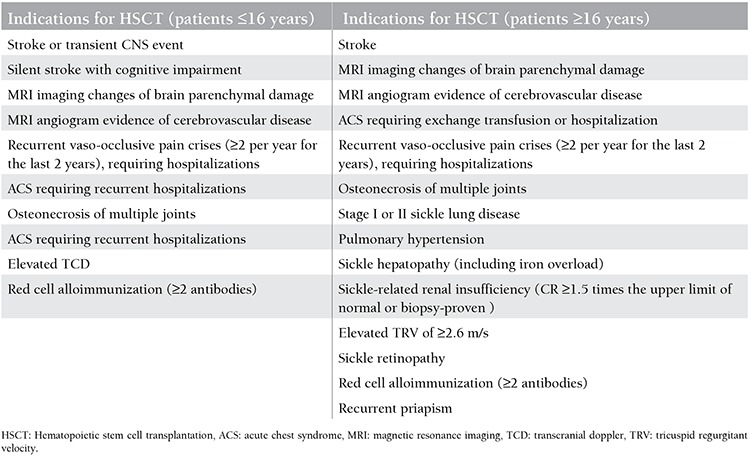
Indications for hematopoietic stem cell transplantation.

**Table 2:**
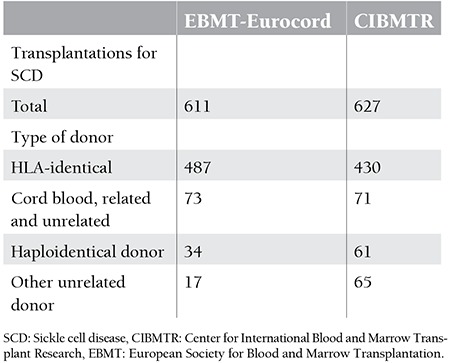
Transplantations for sickle cell disease by donor type and overall survival according to European Society for Blood and Marrow Transplantation-Eurocord and Center for International Blood and Marrow Transplant Research.

**Table 3:**
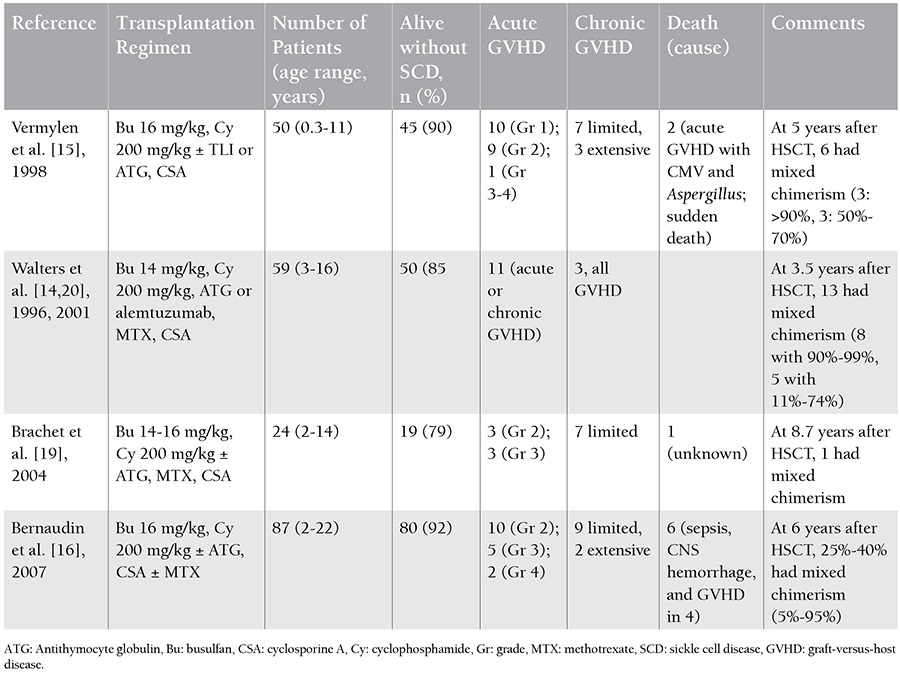
Myeloablative hematopoietic stem cell transplantation with matched related donors.

**Table 4:**
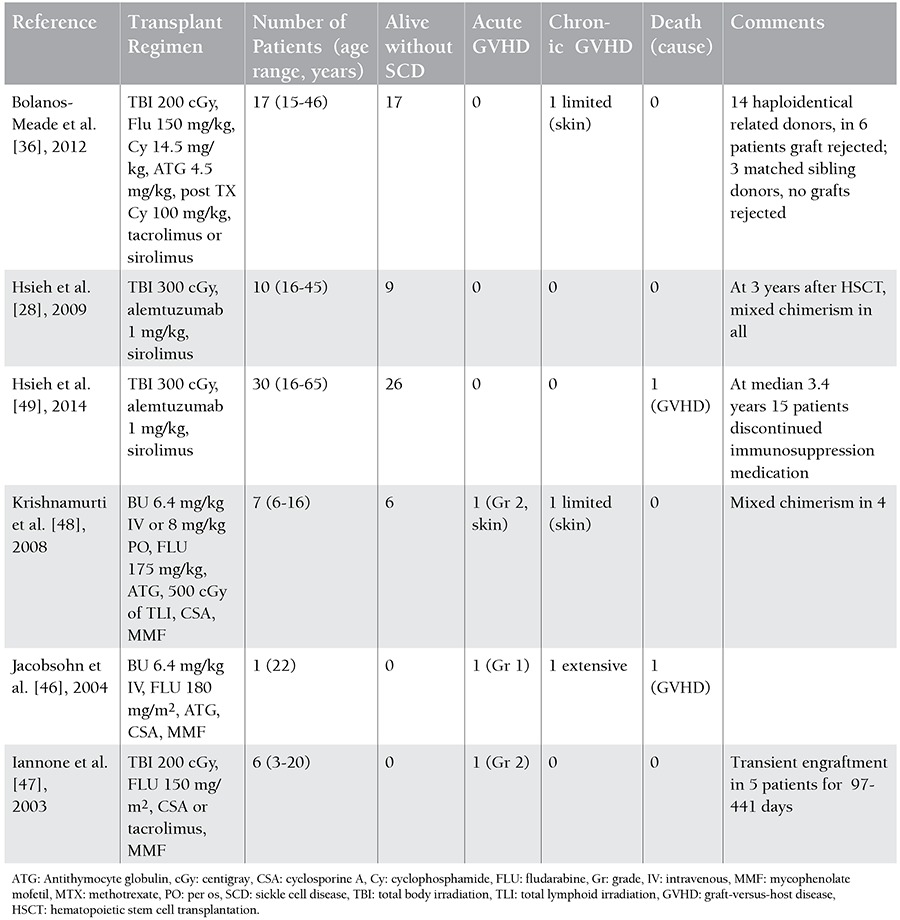
Hematopoietic stem cell transplantation from matched related donors with nonmyeloablative conditioning.
